# Letter to Editor Regarding “Prognostic Impact of Hepatectomy Versus Radiofrequency Ablation for Non‐Small Hepatocellular Carcinoma (2–3 Cm): A Case‐Matched Study”

**DOI:** 10.1002/ags3.70075

**Published:** 2025-08-06

**Authors:** Changzhi Chen, Jieyun Cai, Jinfeng Qiu, Lin Li

**Affiliations:** ^1^ Department of Hepatobiliary and Pancreatic The Third Affiliated Hospital of Guang Xi Medical University Nanning China; ^2^ Zhujiang Hospital Southern Medical University Guangzhou China

**Keywords:** hepatectomy, hepatocellular carcinoma, radiofrequency ablation

We read with interesting the study by Yuki Kitano et al. [1], this study on hepatocellular carcinoma (HCC) measuring 2–3 cm (≤ 3 nodules) demonstrates that compared to radiofrequency ablation (RFA), hepatectomy significantly reduces local recurrence rates and shows a trend toward improved overall survival (OS) and recurrence‐free survival (RFS) after propensity score matching. The findings suggest that for such patients, hepatectomy should be prioritized when liver function and tumor location permit, while alpha‐fetoprotein (AFP) levels may serve as a key indicator for individualized treatment decisions.

In clinical practice, the decision to perform radiofrequency ablation (RFA) is influenced by both liver function and tumor location. For patients with poor liver function, clinicians often choose RFA, however, when a tumor lies on the liver surface or is adjacent to other organs, concerns about bleeding, tumor dissemination, or injury to surrounding structures frequently lead to selection of hepatectomy or laparoscopic ablation. When the tumor is located near major blood vessels or deep within the liver, extensive surgical damage or large liver resection may be required. Due to concerns about the liver function of some patients, ablation is often chosen as the preferred treatment. The study has mitigated the impact of poor liver function through Propensity Score Matching (PSM). However, proximity of the tumor to blood vessels often compromises the ablation zone and effectiveness of RFA [2]. Furthermore, while the RFA ablation margin in the study was 5 mm, surgical resection margins typically exceed this range [3]. This discrepancy may explain the higher local recurrence rate observed in the study.

## Author Contributions

**Changzhi Chen:** writing – original draft, conceptualization, writing – review and editing. **Jieyun Cai:** conceptualization, writing – review and editing. **Jinfeng Qiu:** writing – review and editing. **Lin Li:** writing – review and editing.

## Conflicts of Interest

The authors declare no conflicts of interest.

## Linked Articles

This article is linked to Kitano et al. paper. To view this article, visit https://doi.org/10.1002/ags3.70046.
